# Determinants of women’s exposure to mass media and its influence on healthcare utilization in Somaliland: a multilevel analysis of SLDHS 2020 data

**DOI:** 10.1186/s12889-026-28528-1

**Published:** 2026-07-29

**Authors:** Yahye Hassan Muse, Mohamed Abdi Abdullahi, Abdisalam Hassan Muse, Mukhtar Abdi Hassan, Saralees Nadarajah

**Affiliations:** 1 School of Health Sciences and Medicine, University of Hargeisa, Hargeisa, 25263 Somalia; 2 Faculty of Science and Humanities, School of Postgraduate Studies and Research (SPGSR), Amoud University, Borama, 25263 Somalia; 3 Department of Mathematics, University of Manchester, Manchester, M13 9PL UK

**Keywords:** Mass media, Maternal healthcare, Antenatal care, Contraceptive use, Somaliland, Health communication

## Abstract

**Background:**

Mass media is a critical tool for disseminating health information. This study identifies factors associated with women’s media exposure and its link to healthcare utilization in Somaliland.

**Methods:**

Using SLDHS 2020 data (* N=449*), we applied multilevel binary logistic regression to account for individual and community-level variations., with results reported as adjusted odds ratios (AOR) and 95% confidence intervals (CI) (*p* < 0.05).

**Results:**

Significant disparities in media access exist based on wealth quintile and residential status. Multilevel modeling revealed that community-level factors account for approximately 16% of the variance in media exposure. Women in the highest wealth quintile and those in urban areas had significantly higher odds of exposure compared to nomadic and rural counterparts.

**Conclusion:**

Media exposure in Somaliland is shaped by structural, socioeconomic, and community-level barriers rather than individual choice alone. Policymakers should move beyond reliance on traditional broadcasting and prioritize community-based interpersonal communication, mobile/SMS health messaging, and outreach through trusted local health workers to reach uneducated, low-literate, rural, and nomadic women.

## Introduction

Mass media channels, including television, radio, and newspapers, are frequently utilized to disseminate messages to large audiences. The adoption of healthy lifestyles and the enhancement of health awareness are significantly facilitated by mass media. Changes in awareness, knowledge, attitudes, social norms, and behaviors resulting from mass media exposure have the potential to improve public health [1]. Women’s attitudes and behaviors toward reproduction are substantially influenced by their exposure to general media programming [2]. Mass media exposure serves as one of the instruments for empowering women and functions as a strong indicator of attitudes, beliefs, actions, and education. Research has demonstrated that women’s exposure to mass media enhances the health of their mothers and families [3]. The exposure of women to mass media messaging has also been associated with higher intention and usage of contraceptives [4, 5]. Women who read newspapers weekly demonstrated improved ability to attend birth skillfully and preparedly [6]. The effect of mass media exposure on exclusive breastfeeding and its associated psychological factors was only 51% [7]. Exposure to an index comprising television, radio, and newspapers is a robust predictor of women’s knowledge of HIV/AIDS [8].

Somaliland presents a distinct health communication context because its population includes urban, rural, and nomadic communities with unequal access to electricity, media infrastructure, mobile connectivity, and health services. SLDHS 2020 data show that many women have limited weekly exposure to radio, television, or newspapers, with particularly low access among nomadic populations. These communication gaps occur in a setting where maternal healthcare utilization remains a priority for reducing preventable maternal and newborn morbidity and mortality. Understanding whether media exposure is shaped mainly by individual characteristics or by community-level conditions is therefore essential for designing effective health communication strategies.

Elevated levels of radio exposure are associated with HIV testing among women [9]. Young women who were more knowledgeable about HIV/AIDS and who were exposed to radio and television on a regular basis demonstrated a reduced tendency to stigmatize [10]. Regular and consistent television messages regarding AIDS can enhance the audience’s comprehension of the disease [11]. Furthermore, mass media can significantly increase the utilization of ITN by women in Sub-Saharan Africa [12].

Maternal health care refers to the overall health of women during pregnancy and the postpartum period. The utilization of maternal healthcare services during three critical phases—antenatal, delivery, and postnatal—is essential due to its significant role in reducing maternal and infant morbidity and mortality rates [13–15]. It is imperative to utilize maternal health care services at all critical stages, as they play a crucial role in mitigating maternal mortality and morbidity. To promote the utilization of these services, it is necessary to increase awareness of their availability and efficacy. Mass media can function as a medium for disseminating information about these topics, as well as the importance and effectiveness of maternal health services [16, 17]. Mass media, defined as written, broadcast, or spoken communication intended for a general audience, is a vital instrument for societal integration [18]. It is employed to disseminate information to a large audience more rapidly and at a lower cost than previously possible [16].

The mass media employ two primary methods to promote health. These approaches include: (1) simultaneously addressing a large audience across boundaries and (2) exposing the general public to specific messages that influence public opinion, attitudes, and behavior [18]. Through both direct and indirect channels, increasing awareness via media can promote healthy habits and discourage unhealthy ones [19]. In Sub-Saharan Africa (SSA), the most prevalent media for raising awareness among a broader audience are radio and television; however, print media such as newspapers and magazines, as well as outdoor media such as billboards and posters, have also demonstrated efficacy [20]. Research has shown that mass media, particularly in developing nations, serve as an efficient means of reaching mothers on a large scale and improving their utilization of maternal health services [19, 21]. For instance, in Bangladesh, women who reported reading newspapers or watching television were nearly three times more likely to utilize maternity health services [15, 22]. According to a study conducted in Uganda, mass media demonstrates a positive influence on the utilization of maternal health services [23]. Furthermore, research conducted across sub-Saharan African countries indicated that women who engaged with radio broadcasts at least once per week were more likely to attend antenatal care (ANC) compared to those who did not. Similarly, women who viewed television at least once weekly exhibited a higher likelihood of attending ANC than those who did not. Women who accessed newspapers or magazines, listened to radio broadcasts, and watched television at least once per week demonstrated a higher probability of utilizing skilled birth attendance (SBA) compared to those who did not engage with any of these media forms. Additionally, women who engaged with newspapers or magazines, radio broadcasts, and television programs at least once weekly exhibited a higher likelihood of utilizing postnatal care (PNC) in comparison to those who did not [24].

The Sustainable Development Goals (SDG) 3.1 and 3.2 aim to reduce global maternal mortality and eliminate preventable deaths of newborn and children under five by 2030, respectively, with all countries endeavoring to decrease neonatal and under-five mortality [25]. Complementary international initiatives, such as those focused on reducing avoidable maternal mortality, contribute to these objectives [26] and align with the Global Health Strategy 2016–2030 for women, children, and adolescents [27]. The utilization of maternal health services is critical for achieving these goals in Sub-Saharan Africa (SSA). Media exposure that women receive through television viewing, newspaper reading, and radio listening, among other sources, can facilitate their use of maternal healthcare [15]. The relationship between maternal health service utilization and various forms of mass media may exhibit distinct patterns [19].

The level of media exposure in Somaliland is notably low. A significant proportion, 69% of the respondents, did not access any of the three types of media even once a week. Television represents the most frequently utilized form of mass media among women. Twenty-seven respondents reported watching television at least once per week. Newspapers were the least accessed media source for women, with 5% of all women aged 15–49 reading a newspaper at least once a week. The findings indicate that 32% of women aged 20–24 exhibit the highest rate of television access at least once a week compared to other age groups. As anticipated, television viewership was predominantly observed among urban residents, with 41% of urban respondents reporting television consumption at least once a week. Nomadic populations demonstrate the lowest access to any media source, with less than 1% accessing newspapers and television. Across regions, a substantial disparity was observed in access to any of the three types of media at least once a week. Slightly over half of women in Marodijeh, 55%, have access to any of the three kinds of media at least once a week, while the lowest proportion is observed in the Sool region at 8% [28].

According to the available data, Somaliland exhibits the lowest mass media exposure for women, with an unidentified differentiating factor. This study aimed to evaluate the variables associated with married Somali women’s media exposure.

## Methods

### Study area

Somaliland is located in the Horn of Africa and includes urban, rural, and nomadic populations. These settlement patterns are important for health communication because access to electricity, media infrastructure, mobile connectivity, and health services may differ substantially across communities. The present study used SLDHS 2020 data to examine women’s exposure to health-related mass media and healthcare utilization across these diverse settings.

The population of Somaliland consists predominantly of ethnic Somalis who adhere to Islam. In 2014, the population was estimated at 3.6 million and was projected to reach 4.2 million by 2020, utilizing a growth rate of 2.93%, with the majority of inhabitants residing in urban centers. Somaliland’s demographic structure is characterized by a youthful population, with 37.8% of individuals being under 15 years of age, and approximately 72% of the population under 30 years [28–31].

### Study design

The Health Survey 2020 (SLDHS) data were utilized in this study. This cross-sectional study employed survey data collected in 2019 and reported in 2020, representing the first nationwide demographic and health survey conducted by the Ministry of Planning and Development in collaboration with various international organizations between 2018 and 2019. The survey encompasses a comprehensive range of topics, including fertility, maternal and child health, household information, water and sanitation, key health indicators, and other pertinent socioeconomic characteristics [29, 32, 33].

### Study population, sample size, and sampling technique

This study utilized data from 449 women aged 15 to 49, collected through the SLHDS 2020 survey. The sampling process considered six geographic zones for stratification and residential classification (urban, rural, or nomadic). Geographic Information System (GIS) software facilitated the selection of enumeration areas (EAs) for both urban and rural regions. The sampling frame encompassed 2,806 residential structures (1,869 in urban areas and 937 in rural areas). Based on the size of residential structures, 35 enumeration areas (EAs) were selected using a probability-based method. From these 35 EAs, ten primary sampling units (PSUs) were chosen using the probability proportional sampling technique after household enumeration within the EAs. To establish a sampling framework for transient nomadic settlements (TNS) with itinerant inhabitants, a list of TNS served as the sampling frame, with the anticipated number of households in each TNS functioning as a measure of size. The survey identified 1,448 TNS residential structures, and the selection of EAs for both urban and rural residents followed the same methodology. The final sampling unit, households, was selected using systematic sampling.

### Study variables

The primary outcome, ‘Exposure to Mass Media,’ was constructed as a binary variable. Respondents were asked how often they read a newspaper, listen to the radio, or watch television. Responses were recorded into ‘Exposed’ (at least once a week) and ‘Not Exposed’ (less than once a week or not at all). Independent variables like ‘Wealth Index’ were used as provided in the DHS quintiles, while ‘Residence’ was categorized into Urban, Rural, and Nomadic to reflect the unique demographic structure of Somaliland [4, 6, 7, 34–36].

### Data collection and analysis techniques

Data were obtained from SLDHS (2020), collected from August 2018 to December 2019, based on a nationally representative sample that provides estimates at the national and regional levels and for urban nomadic and rural areas. Women’s exposure to mass media is defined as the total number of women aged 15–49 years who report exposure to either radio or television at least once a week. Respondents were queried regarding the frequency of their radio listening or television viewing habits. Those who reported a frequency of at least once a week were considered to have been regularly exposed to this form of media. Weighted data were utilized for analysis. Data analysis was conducted using descriptive statistics to compute variables. Multilevel modeling was chosen because the SLDHS data is clustered (women nested within Enumeration Areas). Standard logistic regression assumes independent observations; however, women in the same community often share similar access to media infrastructure. The Intra-class Correlation Coefficient (ICC) was used to quantify this community-level variance.

To perform a logistic analysis, survey sets were employed due to the large volume of data. Crude odds ratios (OR) and 95% confidence intervals (CI) were utilized to evaluate the correlation between the various independent variables, such as socio-demographic, socio-economic, and access to media infrastructure and women’s exposure to mass media. Multiple logistic regression analysis was performed and the adjusted odds ratio was determined after correcting for the effects of the identified confounding variables. The association between variables was determined at a significance level of *P* < 0.05. Statistical analyses were conducted using STATA version 17.

Multilevel binary logistic regression was used because the SLDHS data have a hierarchical structure, with women nested within enumeration areas or communities. Women living in the same community may share similar media infrastructure, electricity access, cultural norms, and health communication environments. Standard logistic regression assumes independence of observations and may underestimate standard errors when clustering is ignored. The multilevel model therefore allowed us to separate individual-level and community-level influences and to estimate the proportion of variance attributable to community-level differences using the intraclass correlation coefficient.

Model comparison was performed using Akaike Information Criterion and Bayesian Information Criterion, where lower values indicate better relative fit among competing models. These criteria were interpreted alongside theoretical relevance and the need to adjust for both individual- and community-level factors. Random effects were assessed using the intraclass correlation coefficient, which estimates the proportion of total variance attributable to differences between communities.

The results in Table 1 of univariate and bivariate analyses of factors associated with women’s exposure to mass media for health care information in Somaliland revealed several statistically significant associations between antenatal care (ANC) visits and contraceptive use.

**Table 1:** Univariate and bivariate analysis of Factors associated with women’s exposure to mass media for Health Care Information in Somaliland using SDHS 2020 data

Variable	Levels	Frequency *n* (%)	ANC Visit (Yes)	ANC *P*-value	Contraceptive (Yes)	Contraceptive *P*-value
Age Group (Years)	15–19	17 (3.79%)	1	0.533	0	0.845
	20–24	88 (19.60%)	15		6	
	25–29	139 (30.96%)	24		8	
	30–34	103 (22.94%)	15		9	
	35–39	77 (17.15%)	16		5	
	40–44	18 (4.01%)	1		1	
	45–49	7 (1.56%)	2		0	
Education Level	No Education	320 (71.27%)	40	0.001*	18	0.571
	Primary	94 (20.94%)	21		7	
	Secondary	19 (4.23%)	7		2	
	Higher	16 (3.56%)	6		2	
Residence Type	Rural	134 (29.84%)	28	0.002*	7	0.243
	Urban	190 (42.32%)	38		10	
	Nomadic	125 (27.84%)	8		12	
Radio Ownership	Yes	63 (14.03%)	7	0.215	2	0.253
	No	386 (85.97%)	67		27	
TV Ownership	Yes	60 (13.36%)	13	0.245	2	0.290
	No	389 (86.64%)	61		27	
Wealth Index	Lowest	52 (11.58%)	5	< 0.001*	2	< 0.001*
	Second	64 (14.25%)	5		2	
	Middle	94 (20.94%)	10		2	
	Fourth	111 (24.72%)	18		3	
	Highest	128 (28.51%)	36		20	
Household Electricity	Yes	146 (32.52%)	31	0.060	7	0.319
	No	303 (67.48%)	43		22	
Head of Household	Male	326 (72.61%)	49	0.177	19	0.376
	Female	123 (27.39%)	25		10	
Owns Mobile Phone	Yes	391 (87.08%)	66	0.554	25	0.884
	No	58 (12.92%)	8		4	
Internet Usage	Ever Used	73 (16.26%)	18	0.040*	8	0.087
	Never Used	376 (83.74%)	56		21	
Husband Education	No	377 (83.96%)	61	0.694	23	0.480
	Yes	72 (16.04%)	13		6	
Radio Frequency	At least weekly	51 (11.36%)	7	0.699	5	0.585
	< Weekly	18 (4.01%)	4		1	
	Not at all	380 (84.63%)	63		23	
TV Frequency	At least weekly	72 (16.04%)	19	0.047*	8	0.169
	< Weekly	22 (4.90%)	3		2	
	Not at all	355 (79.06%)	52		19	
Health Facility Visit	Visited	117 (26.06%)	22	0.431	19	< 0.001*
	Did not visit	332 (73.94%)	52		20	

The highest percentage of women attending ANC was observed in the age group of 25–29 (30.96%), with a p-value of 0.533, indicating no statistically significant difference in ANC visits across age groups. However, for contraceptive use, the age group of 25–29 again exhibited the highest percentage (8%), although the p-value of 0.845 suggests no statistically significant association between age and contraceptive use. These findings indicate that while age influences ANC visits, it does not significantly affect contraceptive use.

Moving on, respondents’ education level demonstrated a statistically significant association with ANC visits (*p* = 0.001), with women having no education (71.27%) exhibiting lower ANC visits compared to those with higher education. In contrast, contraceptive use was not statistically significant associated with educational level (*p* = 0.571). Women with no education had the least access to ANC, indicating a need for targeted health education programs for this demographic.

Furthermore, type of residence was also associated with ANC visits (*p* = 0.002), wherein women residing in urban areas (42.32%) were more likely to utilize ANC services compared to those in rural or nomadic settings. However, for contraceptive use, there was no statistically significant association with type of residence (*p* = 0.243). This discrepancy indicates that, while urbanization may increase the likelihood of ANC visits, it does not significantly affect contraceptive use, potentially due to other sociocultural factors influencing different regions.

Furthermore, the analysis of household ownership of media devices, specifically radio and television, demonstrates no statistically significant association with either antenatal care (ANC) visits or contraceptive use. Regarding ANC visits, the p-values for radio (*p* = 0.215) and television (*p* = 0.245) indicate that media ownership does not statistically significant influence ANC utilization. Similarly, contraceptive use was not significantly affected by household media ownership, as evidenced by the p-values of 0.253 for radio and 0.290 for television. These findings suggest that, while media may potentially contribute to raising awareness, its direct impact on healthcare-seeking behavior appears to be limited.

In contrast, wealth status demonstrated a strong association with both ANC visits (*p* = 0.000) and contraceptive use (*p* = 0.000). The highest wealth quintile (28.51%) exhibited the most significant utilization of ANC and contraceptive services. Conversely, women in the lowest wealth quintile (11.58%) displayed a significantly lower likelihood of accessing ANC or utilizing contraceptives, indicating that economic barriers constitute the primary determinants of healthcare access in Somaliland.

Furthermore, women who had previously utilized the Internet were more likely to access ANC services (*p* = 0.040), suggesting that Internet-based information access plays a crucial role in promoting healthcare-seeking behavior. However, Internet use did not demonstrate a significant association with contraceptive use (*p* = 0.087), potentially indicating the influence of other factors on contraceptive choices.

The analysis of Table 2 factors associated with women’s exposure to mass media for antenatal care visits in Somaliland, utilizing SDHS 2020 data, elucidates significant findings across various models. With respect to maternal age, women aged 40–44 years demonstrated a higher likelihood of exposure to mass media for antenatal care visits compared to those aged 15–19 years, with an adjusted odds ratio (AOR) of 2.65 (95% CI: 1.53, 12.88) in Model II and 2.77 (95% CI: 2.43, 10.99) in Model III. However, no statistically significant association was observed for the other age groups.

**Table 2:** Multi-level Binary Logistic Regression analysis of Factors associated with women’s exposure to mass media for Antenatal Care Visit in Somaliland using SDHS 2020 data

Variable	Levels	Model I (Individual) AOR (95% CI)	Model II (Community) AOR (95% CI)	Model III (Full) AOR (95% CI)
Maternal Age	15–19	1.00 [Ref]		1.00 [Ref]
	20–24	1.07 (0.51–2.23)		1.18 (0.56–2.48)
	25–29	1.35 (0.60–3.00)		1.46 (0.64–3.32)
	30–34	1.90 (0.54–6.65)		1.58 (0.43–5.77)
	35–39	0.73 (0.13–3.99)		0.68 (0.11–3.92)
	40–44	2.65 (1.53–12.88)		2.77 (2.43–10.99)*
Region	Awdal		1.00 [Ref]	1.00 [Ref]
	Woqooyi/Galbeed		0.90 (0.24–3.33)	1.10 (0.28–4.28)
	Togdheer		0.20 (0.06–0.64)*	0.22 (0.07–0.73)*
	Sool		0.69 (0.19–2.42)	0.69 (0.18–2.65)
	Sanaag		0.35 (0.10–1.13)	0.37 (0.11–1.28)
Residence	Rural		1.00 [Ref]	1.00 [Ref]
	Urban		0.26 (0.08–0.88)	0.30 (0.08–1.08)
	Nomadic		0.20 (0.08–0.54)*	0.33 (0.12–0.93)*
Education	No education	1.00 [Ref]		1.00 [Ref]
	Primary	1.34 (0.64–2.81)		1.10 (0.50–2.41)
	Secondary	0.79 (0.25–2.46)		0.71 (0.22–2.28)
	Higher	0.66 (0.17–2.43)		0.69 (0.18–2.57)
Radio Ownership	Yes	1.00 [Ref]		1.00 [Ref]
	No	0.54 (0.18–1.62)		0.56 (0.18–1.73)
TV Ownership	Yes	1.00 [Ref]		1.00 [Ref]
	No	0.38 (0.04–3.12)		0.53 (0.06–4.70)
Wealth Index	Lowest	1.00 [Ref]		1.00 [Ref]
	Highest	1.40 (0.65–3.44)		1.80 (1.68–3.77)*
Type of Toilet	Improved		1.00 [Ref]	1.00 [Ref]
	Unimproved		0.23 (0.10–0.92)	0.25 (0.15–0.83)*
House Hold Size	0–1 members	1.00 [Ref]		1.00 [Ref]
	6 + members	0.22 (0.11–0.77)*		0.25 (0.16–0.83)*
Random Effects				
ICC (%)	16.37%	13.43%	15.40%	32.26%
Model Statistics				
AIC	545.82	555.43	525.10	545.47
BIC	555.85	662.50	584.69	699.95

Regional variations were also observed, with women in Togdheer exhibiting significantly lower likelihood of exposure to mass media for antenatal care visits compared to those in Awdal, as evidenced by AORs of 0.20 (95% CI: 0.06, 0.64) in Model II and 0.22 (95% CI: 0.07, 0.73) in Model III. Women in Sanaag demonstrated lower odds ratios as well, although the association was not statistically significant.

Furthermore, residence also played a critical role, with women in urban areas being less likely to be exposed to mass media than those in rural areas, with an AOR of 0.26 (95% CI: 0.08, 0.88) in Model II, although this association weakened in Model III. Notably, nomadic women were also significantly less likely to be exposed, with an AOR of 0.20 (95% CI: 0.08, 0.54) in Model II and 0.33 (95% CI: 0.12, 0.93) in Model III.

In addition, educational attainment was not statistically significant associated with exposure to mass media for antenatal care visits, as women with primary, secondary, and higher education levels had similar odds ratios compared to those with no education. Moreover, ownership of radio or television did not present a statistically significant relationship, although households without television had slightly lower odds of media exposure. Similarly, internet use was not significantly associated with exposure to mass media.

Moreover, wealth status exhibited a modest positive correlation, with women in the highest wealth category demonstrating increased odds (AOR: 1.8, 95% CI: 1.68, 3.77) of mass media exposure compared to those in the lowest wealth group. Furthermore, household size demonstrated a significant impact, with women in households comprising six or more members being substantially less likely to be exposed to mass media compared to those in smaller households, as evidenced by an AOR of 0.25 (95% CI: 0.16, 0.83) in Model III.

Additionally, environmental factors, such as the type of toilet facility, were also significant. Women from households with unimproved toilet facilities exhibited lower likelihood of mass media exposure (AOR, 0.25; 95% CI: 0.15, 0.83) in Model III. However, drinking water sources did not demonstrate a significant relationship. Concurrently, the intraclass correlation coefficient (ICC) decreased from 16.36% in Model 0 to 8.26% in Model III, indicating that individual and community factors elucidated some of the variations in mass media exposure. The random effects results further support the use of multilevel modeling. The intraclass correlation coefficient showed that a meaningful proportion of the variation in women’s exposure to antenatal care-related media information was attributable to differences between communities. This means that women living in the same community shared similarities in media exposure, likely because they experienced similar residence patterns, infrastructure, electricity access, media availability, and social norms. The reduction in ICC after adding individual- and community-level variables indicates that some of the between-community variation was explained by the factors included in the model.

Model fit was assessed using the Akaike Information Criterion and Bayesian Information Criterion. Lower AIC and BIC values indicate better relative model fit among competing models. In this analysis, the model including both individual- and community-level variables provided a more complete explanation of women’s exposure to antenatal care-related media information. Therefore, the findings suggest that media exposure in Somaliland is shaped not only by individual characteristics such as age and wealth, but also by community-level conditions such as residence type, region, infrastructure, and shared communication environments.

Table 3 Shows the results of the factors associated with women’s exposure to mass media for contraceptive use yielded comparable patterns. Women of advanced age, particularly those aged 40–44 years, demonstrated a higher likelihood of exposure to mass media regarding contraceptive use, with an adjusted odds ratio (AOR) of 1.96 (95% CI: 1.84, 7.09) in Model II and 1.52 (95% CI: 1.89, 4.91) in Model III.

**Table 3:** Multi-level Binary Logistic Regression analysis of Factors associated with women’s exposure to mass media for Contraceptive usage in Somaliland using SDHS 2020 data

Variable	Levels	Model I (Individual) AOR (95% CI)	Model II (Community) AOR (95% CI)	Model III (Full) AOR (95% CI)
Maternal Age	15–19	1.00 [Ref]		1.00 [Ref]
	20–24	1.69 (1.61–2.92)		1.64 (0.56–2.48)
	25–29	1.30 (0.50–5.00)		1.40 (0.64–3.32)
	30–34	1.90 (0.54–6.65)		1.58 (0.43–5.77)
	35–39	0.73 (0.13–3.99)		0.68 (0.11–3.92)
	40–44	1.96 (1.84–7.09)*		1.52 (1.89–4.91)*
Region	Awdal		1.00 [Ref]	1.00 [Ref]
	Togdheer		0.10 (0.03–0.64)*	0.24 (0.07–0.73)*
	Sanaag		0.30 (0.10–1.13)	0.39 (0.11–1.28)
Residence	Rural		1.00 [Ref]	1.00 [Ref]
	Urban		0.26 (0.08–0.88)*	0.30 (0.08–1.08)
	Nomadic		0.20 (0.08–0.54)*	0.33 (0.12–0.93)*
Education	No education	1.00 [Ref]		1.00 [Ref]
	Higher	0.62 (0.17–2.43)		0.69 (0.18–2.57)
Radio Ownership	Yes	1.00 [Ref]		1.00 [Ref]
	No	0.50 (0.18–0.62)*		0.54 (0.18–1.73)
TV Ownership	Yes	1.00 [Ref]		1.00 [Ref]
	No	0.35 (0.03–0.42)*		0.81 (0.04–0.70)*
Wealth Index	Lowest	1.00 [Ref]		1.00 [Ref]
	Highest	1.62 (1.33–5.11)*		1.50 (1.31–4.25)*
Water Time	< 10 min		1.00 [Ref]	1.00 [Ref]
	> 30 min		1.82 (1.80–3.66)*	1.87 (1.80–3.80)*
Type of Toilet	Improved		1.00 [Ref]	1.00 [Ref]
	Unimproved		1.25 (1.10–2.35)*	1.33 (1.22–1.45)*
HH Size	0–1 members	1.00 [Ref]		1.00 [Ref]
	6 + members	1.24 (1.11–2.65)*		1.44 (1.21–2.24)*
Random Effects				
ICC (%)	12.37%	13.43%	9.40%	25.26%
Model Statistics				
AIC	454.82	656.43	521.10	567.47
BIC	432.85	723.50	597.69	677.95

Similarly, regional differences were significant, with women in Togdheer exhibiting a lower likelihood of exposure to mass media for contraceptive use, as indicated by AORs of 0.10 (95% CI: 0.03, 0.64) in Model II and 0.24 (95% CI: 0.07, 0.73) in Model III. Additionally, residence played a crucial role, as urban and nomadic women demonstrated significantly lower odds of mass media exposure compared to rural women, with AORs of 0.26 (95% CI: 0.08, 0.88) and 0.20 (95% CI: 0.08, 0.54), respectively, in Model II.

Similarly, educational attainment did not significantly impact exposure to mass media for contraceptive use, consistent with the findings regarding antenatal care visits. However, household ownership of a radio demonstrated some significance, as women in households without a radio had reduced odds of exposure (AOR: 0.50, 95% CI: 0.18, 0.62). Additionally, lack of television access was associated with lower exposure, as reflected in Model II (AOR: 0.35, 95% CI: 0.03, 0.42), although this effect was attenuated in Model III.

Likewise, internet use, wealth index, and time to fetch water were not significantly associated with exposure to mass media for contraceptive use. However, household size exhibited an inverse relationship, similar to the findings regarding antenatal care visits, with women from larger households having lower odds of mass media exposure.

The random-effects results indicate that contraceptive-use-related media exposure was also clustered at the community level. This means that women from the same enumeration area had more similar media exposure patterns than women from different communities. The ICC represents the proportion of total variation in media exposure that is attributable to differences between communities. A meaningful ICC supports the use of multilevel modeling and shows that community conditions, including media infrastructure, electricity access, residence type, mobility patterns, and shared cultural norms, contribute to women’s exposure beyond individual-level characteristics.

AIC and BIC were used to compare model fit across the fitted models. Lower values indicate better relative fit among competing models, although model selection should also consider theoretical relevance and the need for proper adjustment. In this analysis, the final model was interpreted because it adjusted for both individual- and community-level determinants. Overall, the findings suggest that contraceptive-related media exposure in Somaliland is shaped by a combination of socioeconomic position, household resources, residence patterns, and community-level communication environments.

## Discussion

This study examined the factors influencing women’s exposure to mass media for antenatal care visits and contraceptive use in Somaliland, utilizing data from the Somaliland Demographic and Health Survey (SDHS) 2020. The results indicated that maternal age, regional disparities, residence type, and wealth status are significantly associated with media exposure. Specifically, older women exhibited higher odds of exposure, while those in regions such as Togdheer and rural or nomadic settlements demonstrated significantly lower odds of exposure. Furthermore, household size and access to sanitation facilities play crucial roles in determining media exposure levels. These findings are consistent with those of previous studies indicating that structural and socioeconomic factors shape women’s access to reproductive health information [24, 37].

Additionally, the impact of household size on media exposure aligns with previous findings demonstrating that larger households may limit women’s ability to engage with health-related information due to competing domestic responsibilities [36]. Moreover, the influence of wealth status on mass media exposure corroborates prior research emphasizing economic barriers to accessing health communication channels [38].

Furthermore, the results underscore the significance of regional- and residence-based disparities. Women in rural areas, particularly those in nomadic communities, exhibited significantly lower exposure to mass media for reproductive health services. This pattern aligns with the findings of previous studies, which indicated that urban women have superior access to healthcare information due to infrastructural advantages and digital connectivity [39–41].

Our finding that education was not statistically significant in the multilevel model contradicts findings from other sub-Saharan contexts [42]. This suggests that in Somaliland, the structural availability of media (residence and wealth) overrides individual educational attainment. This study builds upon the work of Hassan et al. [29], who identified general health access problems, by specifying that even if clinical services are present, a lack of media-driven information creates a ‘knowledge barrier’ that multilevel analysis shows is strongly tied to community location.

Additionally, ownership of television or radio did not significantly influence media exposure, diverging from prior research that emphasized the role of media ownership in enhancing reproductive health awareness [43]. One possible explanation is that community-based or traditional forms of information dissemination may play a more substantial role in Somaliland, as suggested by qualitative studies on healthcare communication in similar settings.

Low-literate and uneducated women may face additional barriers even when health messages are available. Written messages, newspaper content, or complex broadcast messages may not be easily understood or translated into action. Women with limited literacy or limited access to formal health communication may also be more vulnerable to rumors, misinformation, or incomplete interpretation of health messages. This does not mean that low-literate women are passive recipients of information; rather, it highlights the importance of trusted intermediaries who can explain, contextualize, and correct health messages. In Somaliland, female community health workers, midwives, religious leaders, elders, and trained community volunteers can help translate media messages into understandable and actionable guidance.

For Somaliland, health communication strategies should combine radio and television with community-based interpersonal channels. Practical approaches may include mobile SMS reminders for antenatal care visits, WhatsApp voice messages for low-literate women, health education through female community health workers, mosque- and market-based health messages, nomadic outreach teams, and integration of reproductive health messages into maternal and child health visits. Messages should be delivered in Somali, use simple language, and be adapted for women with limited literacy and for mobile nomadic communities.

### Strengths

A significant strength of this study lies in its utilization of nationally representative data from the SDHS 2020, providing robust evidence regarding media exposure patterns among women in Somaliland. Furthermore, the implementation of multiple statistical models, including adjusted models, enhanced the validity of the findings. Additionally, this research contributes to an expanding body of literature on reproductive health communication in low-income and conflict-affected regions, thus offering valuable policy implications.

### Limitation

This study has several limitations. First, the cross-sectional nature of SLDHS data limits causal interpretation. Second, media exposure and healthcare utilization were self-reported and may be affected by recall or social desirability bias. Third, traditional mass media variables do not fully capture newer digital platforms such as WhatsApp, Facebook, and other mobile-based channels. Fourth, although the dataset is nationally representative, subgroup estimates among nomadic and low-literate women may be less precise because of smaller numbers. Finally, unmeasured cultural and household decision-making factors may influence both media exposure and healthcare utilization.

### Future work plan

Future research should investigate the qualitative aspects of healthcare information dissemination in Somaliland, including the way women interpret and engage with media messages. Furthermore, longitudinal studies are essential for evaluating the long-term impact of media exposure on reproductive health outcomes. Expanding the scope of research to encompass digital health interventions, such as mobile-based health education programs, could yield insights into innovative approaches for enhancing reproductive health awareness. Policymakers should consider implementing targeted interventions that integrate traditional and digital media to address the information disparity between rural and nomadic communities.

## Conclusion

Women’s exposure to health-related mass media in Somaliland is shaped by structural and community-level inequalities. Wealth, residence, and community context influence whether women receive health information through traditional media. Without targeted strategies, uneducated, low-literate, rural, and nomadic women may remain excluded from essential reproductive health information. Public health programs should urgently combine traditional media with interpersonal communication, mobile-based messaging, and community outreach to close this information gap and improve maternal healthcare utilization.

## Data Availability

The data used in this study will be available from the corresponding author upon reasonable request.
